# A Study on a Digital Twin Method for Modelling the Diffusion Process of Fault Gases Inside High-Voltage Switchgear Combining Virtual and Physical Models

**DOI:** 10.3390/s26175538

**Published:** 2026-08-31

**Authors:** Xin Wang, Hao Luo

**Affiliations:** 1 Guoneng Guangtou Beihai Power Generation Co., Ltd., Beihai 536017, China; 2 State Key Laboratory of Power Transmission Equipment Technology, School of Electrical Engineering, Chongqing University, Chongqing 400044, China

**Keywords:** digital twin, high-voltage switchgear, gas transport, virtual sensing, physical testing

## Abstract

**Highlights:**

**What are the main findings?**

- A 1:1 digital twin model is constructed to analyse the diffusion behaviour of partial discharge characteristic gases in a 10 kV switchgear.
- Obvious differences in gas migration characteristics of CO, CO_2_, O_3_ and NO are uncovered, and the model achieves concentration prediction error within 10% after physical verification.

**What are the implications of the main findings?**

- The conclusions provide engineering guidance for optimizing the layout of internal gas sensors of high-voltage switchgear.
- The virtual sensing strategy based on digital twin helps realize intelligent operation and maintenance of power switchgear.

**Abstract:**

Monitoring gas generated by internal faults in high-voltage switchgear is crucial for early warning and condition assessment; however, the diffusion process between gas generation and detection is currently widely overlooked, which severely compromises the accuracy of gas-based assessments. Implementing “virtual sensing” for high-voltage switchgear using digital twin technology to supplement data from physical sensors can effectively support digitalised and intelligent operation and maintenance. To this end, taking the characteristic gases of partial discharge as the subject of study, we first constructed a 1:1 scale digital twin model of internal gas diffusion within a 10 kV high-voltage switchgear. Calculations revealed significant differences in the diffusion processes of various gases: CO diffuses rapidly and mixes strongly, making it suitable for early warning; CO_2_ and O_3_ tend to accumulate in lower regions and stagnant zones, making them sensitive to faults in the lower sections; whilst NO reflects the channelling effect within the structure. Subsequently, a test platform comprising a 10 kV high-voltage switchgear unit and a gas detector was established for experimental validation. This revealed the trends in gas concentration changes, response sequences and peak behaviour at different fault locations, thereby verifying the validity of the proposed model. The maximum error in concentration balance was within 10 percent, providing a theoretical basis and engineering reference for the optimised placement of gas sensors in high-voltage switchgear and for fault tracing and localisation.

## 1. Introduction

The digital and intelligent transformation of power equipment is a key task in building a new-generation power system. High-voltage switchgear is a critical primary piece of equipment in distribution systems for the distribution, control, protection and fault isolation of electrical energy, and is widely used in substations, distribution rooms and industrial power supply systems. Switchgear features a compact internal structure comprising components such as busbars, circuit breaker contacts, contact boxes, cable terminations, insulating partitions, earthing switches and secondary terminals. During long-term operation, these components are susceptible to factors such as increased contact resistance, insulation ageing, localised moisture ingress, electric field distortion, mechanical vibration and thermal stress, which can lead to latent faults such as partial discharge, poor contact, overheating and insulation degradation. If such defects are not detected promptly, they may further develop into phase-to-phase short circuits, insulation breakdown or even switchgear explosions, posing a serious threat to the safe and stable operation of distribution networks [1,2,3,4]. However, due to limited internal space, it is difficult to implement distributed sensor monitoring; therefore, there is an urgent need to accelerate the digital transformation of high-voltage switchgear to support smart operation and maintenance.

Currently, methods for condition monitoring of high-voltage switchgear primarily include infrared temperature measurement, ultrasonic testing, transient earth voltage detection, ultra-high-frequency (UHF) detection, the pulse current method, and partial discharge detection [4,5,6,7,8]. These methods can reveal abnormal conditions in the equipment from thermal, electrical and acoustic perspectives; however, in actual operating environments, they are susceptible to the influence of cabinet shielding, on-site electromagnetic interference, propagation path attenuation and sensor mounting positions. In contrast, detection methods based on characteristic gases, which originate from air decomposition, insulation-material degradation and oxidation reactions induced by electrical or thermal faults, can reflect the cumulative chemical effects of fault development. Such methods offer strong resistance to electromagnetic interference and are suitable for online monitoring and early warning. Recent advances have further extended gas-sensing research toward computationally assisted sensor-material design. For example, Cui et al. combined density functional theory and machine learning to accelerate screening of WX_2_ (X = S, Se, and Te) materials for detecting C_4_F_7_N decomposition species, demonstrating the potential of data-driven approaches to improve gas-sensor performance [9]. Nevertheless, existing studies have mainly focused on characteristic-gas generation mechanisms, sensing materials and detection methods, and concentration-based criteria, whereas the transport process between the fault source and the physical sensor within the complex internal space of switchgear has received comparatively limited attention.

In this study, a digital twin is defined as a physics-based virtual representation that is geometrically consistent with the physical switchgear and is constrained and validated by sensor measurements. Physical sensors provide gas-concentration time series and environmental information at a limited number of accessible positions. In contrast, the numerical model reconstructs the unmeasured spatiotemporal concentration field and generates corresponding “virtual sensor” responses. The physical measurements in the present work are used primarily for model validation rather than for continuous online model updating. Once the model reliability has been established, the measured and virtual transient responses can be jointly interpreted to support sensor-layout optimisation, early warning and preliminary fault-source localisation. Thus, the digital twin serves as the bridge between limited physical sensing and full-field fault-gas diagnosis rather than as a replacement for physical sensors.

Recent applications of digital-twin technology to switching equipment have increasingly combined high-fidelity virtual models with sparse physical measurements. Yan et al. used digital virtual models of gas-insulated switchgear (GIS) to augment limited partial-discharge (PD) data for condition assessment and source localisation [10,11]. Ding et al. developed a thermal–electric-field digital-twin diagnosis model for high-voltage switchgear by integrating simulation, experimental and operational data [12]. More recently, Wang et al. reconstructed the full temperature field of GIS from sparse sensor measurements with uncertainty quantification for digital-twin applications [13]. These studies demonstrate the value of digital twins for data augmentation, full-field reconstruction and intelligent diagnosis; however, they mainly focus on electrical/thermal states or PD signal features. Physics-based digital-twin studies that directly connect fault-gas transport, multi-point gas measurements and source-related transient-response characteristics in air-insulated switchgear remain limited. This gap motivates the present work.

With the advancement of computational fluid dynamics (CFD), a large number of researchers have adopted CFD methods to analyse gas diffusion patterns; however, these studies typically focus on the visualisation of concentration fields or the response at a single measurement point. Research into the sensitivity to different monitoring point layouts, differences in responses across multiple monitoring points, and combined criteria for multiple gases remains insufficient [5,14,15,16,17,18]. Furthermore, due to the confined spaces, complex structures and general lack of forced ventilation within high-voltage switchgear, the diffusion process of fault gases is influenced not only by concentration gradients but also by the combined effects of factors such as molecular diffusion coefficients, gas molecular mass, weak natural convection, gravitational settling, structural obstructions, local stagnation and wall boundary conditions; consequently, modelling and simulation remain relatively complex. Furthermore, whilst existing studies have largely focused on simple gas diffusion simulations, comparative studies involving full-scale equipment testing are rarely reported; consequently, the accuracy of numerical simulations of gas diffusion processes is difficult to guarantee and warrants further investigation.

In summary, this paper addresses the numerical calculation of fault gases within 10 kV high-voltage switchgear by proposing a digital twin method for the internal gas diffusion process that integrates virtual and physical models. Firstly, a simulation model for the transient transport of gas within the high-voltage switchgear was established, enabling numerical simulation of the entire internal gas diffusion process. Secondly, a 1:1 full-scale 10 kV high-voltage switchgear test platform was set up for synchronous sampling verification, demonstrating the effectiveness of this method in describing gas diffusion behaviour. Its innovations lie in the following: (1) A transient transport simulation model for fault gases was established, incorporating typical internal structures, upper and lower fault points, and multiple monitoring points, systematically analysing the diffusion stages, migration paths and local accumulation patterns of different gases within the switchgear; (2) Using the 1:1 full-scale 10 kV high-voltage switchgear test platform, the concentration response trends, initial response time, time to peak concentration and equilibrium concentration predicted by the simulation model were verified. This method provides key technical support for distributed virtual sensing, the optimised layout of gas sensors, the determination of optimal monitoring intervals, and the traceability and localisation of gas generation points.

## 2. A Digital Twin Approach to the Gas Transport Process in High-Voltage Switchgear Failures

### 2.1. A Digital Twin Approach to Switchgear Status Combining Virtual and Physical Realms

**Figure 1:**
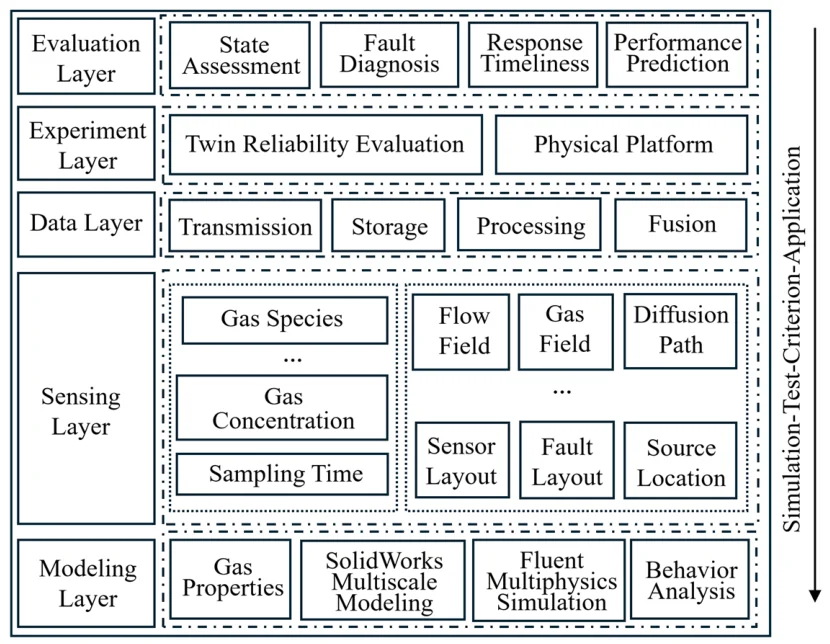
Implementation framework for a virtual–physical hybrid state twin method for switchgear fault detection.

An accurate digital twin forms the foundation for precise digital operation and maintenance of switchgear. In the framework adopted here, the physical layer supplies the actual cabinet geometry, operating conditions and gas-concentration measurements at accessible sampling ports; the modelling layer calculates the transient three-dimensional concentration field; and the reliability-verification layer compares physical measurements with virtual-sensor responses at matched locations and times. Only after this comparison demonstrates acceptable agreement is the numerical model used to infer concentration histories at locations where physical sensors are absent. In this sense, ‘virtual sensing’ denotes model-based estimation of unmeasured gas states constrained by a physically verified model. Based on the virtual–physical fusion method for the insulation-state twin of switchgear [16,19,20,21,22,23], the implementation framework is divided into the modelling and simulation layer, virtual–physical sensing layer, data exchange layer, reliability-verification layer and state-assessment layer. This architecture establishes a transparent relationship among simulation, sensor measurement and subsequent fault diagnosis. This architecture establishes a transparent relationship among simulation, sensor measurement and subsequent fault diagnosis. Figure 1 illustrates the overall virtual–physical implementation framework and the information flow between its functional layers.

### 2.2. Fault Gas Transport Model

To address the decomposition of insulating materials and the transport of characteristic gases caused by electrical and thermal faults within high-voltage switchgear, this paper establishes a mathematical model for the transient migration of multicomponent gases based on computational fluid dynamics (CFD) [24,25,26]. This model comprehensively considers the influence of typical internal structures (busbars, partitions, and contact boxes) on gas flow organisation and component distribution. It employs a set of governing equations centred on the conservation equations for mass, momentum, energy, and components to describe the processes of diffusion, convection, and local accumulation of fault gases within the cabinet’s air environment.

Concerning the description of the flow field, the mixed gas inside the switchgear is treated as a compressible Newtonian fluid, and mass conservation is given by the continuity equation [27]:

(1) $$\frac{\partial \rho }{\partial t}+\nabla \cdot (\rho u)=0$$

Here, ρ is the density of the gas mixture, u is the velocity vector, and *t* is time. The equation of momentum conservation reflects the combined effects of pressure, viscous forces, and thermal buoyancy on the motion of the gas:

(2) $$\frac{\partial }{\partial t}(\rho u)+\nabla \cdot (\rho uu)=-\nabla p+\nabla \cdot \tau +\rho g+F_{th}$$

(3) $$\tau =\mu [(\nabla u)+(\nabla u{)}^{T}]-\frac{2}{3}\mu (\nabla \cdot u)I$$

In the equation, p represents pressure, μ represents dynamic viscosity, τ represents the viscous stress tensor, ρg represents the gravitational term, $F_{th}$ represents the thermal buoyancy term caused by the temperature gradient (which can be solved using the Boussinesq approximation or by coupling the energy equation), and I represents the unit tensor. The energy conservation equation is used to describe the changes in the temperature field caused by local heat generation at the failure site and its feedback effect on the convection field density and the diffusion coefficients of the components:

(4) $$\frac{\partial }{\partial t}(\rho E)+\nabla \cdot \left[u(\rho E+p)\right]=\nabla \cdot (k_{eff}\nabla T)+S_{E}$$

Here, E represents the total energy, $k_{eff}$ represents the effective thermal conductivity, T represents the temperature, and $S_{E}$ represents the heat source term at the fault point (such as heat flux generated by an electric arc or overheating). For multicomponent mixtures, component transport equations are introduced to simulate the transport behaviour of different characteristic gases in air:

(5) $$\frac{\partial }{\partial t}(\rho Y_{a})+\nabla \cdot (\rho uY_{a})=\nabla \cdot (\rho D_{a}\nabla Y_{a})+S_{a}$$

In the equation, $Y_{a}$ represents the mass fraction of the component  a, $D_{a}$ is the diffusion coefficient of that component in air, and $S_{a}$ is the mass production rate per unit volume of the component a at the failure point, which characterises the gas release intensity of the failure source.

To obtain a closed-form solution, appropriate boundary conditions must be applied to the computational domain. The location of the failure point is designated as a mass inlet boundary, and the release rate, component ratio and initial temperature are specified as follows:

(6) $$\left\{\begin{array}{c} {\dot{m}}_{in}={\dot{m}}_{0}\\ Y_{a,in}=Y_{a,0}\\ T_{in}=T_{0} \end{array}\right.$$

where ${\dot{m}}_{0}$ is the gas release rate, $Y_{a,0}$ is the initial mass fraction of the released gas, and $T_{0}$ is the temperature at the fault location. The surfaces of the cabinet walls and the internal solid structures are assumed to be non-slip surfaces and satisfy the conditions of thermal insulation and no mass permeation:

(7) $$\left\{\begin{array}{c} u_{wall}=0\\ \frac{\partial T}{\partial n}=0\\ \frac{\partial Y_{a}}{\partial n}=0 \end{array}\right.$$

In the equation, n represents the direction normal to the outer surface of the wall; the zero normal gradient condition ensures that there is no flux exchange of components or temperature across the wall.

By discretising and solving the above system of equations using the finite volume method, the gas concentration, temperature, and velocity distributions at any point in space and at any given time within the switchgear can be obtained. By combining these simulation results with actual data from flow velocity and temperature sensors within the switchgear, a multi-physics digital twin model of the switchgear can be constructed. This provides crucial virtual sensor data for the early warning of fault gases, the tracking of diffusion paths and the tracing of gas generation sources, thereby supporting equipment condition assessment and intelligent operation and maintenance decision-making.

## 3. Virtual Sensing Analysis of Fault Gas Transport Processes Within Switchgear

### 3.1. Full-Scale Modelling, Simulation and Testing Methods

**Figure 2:**
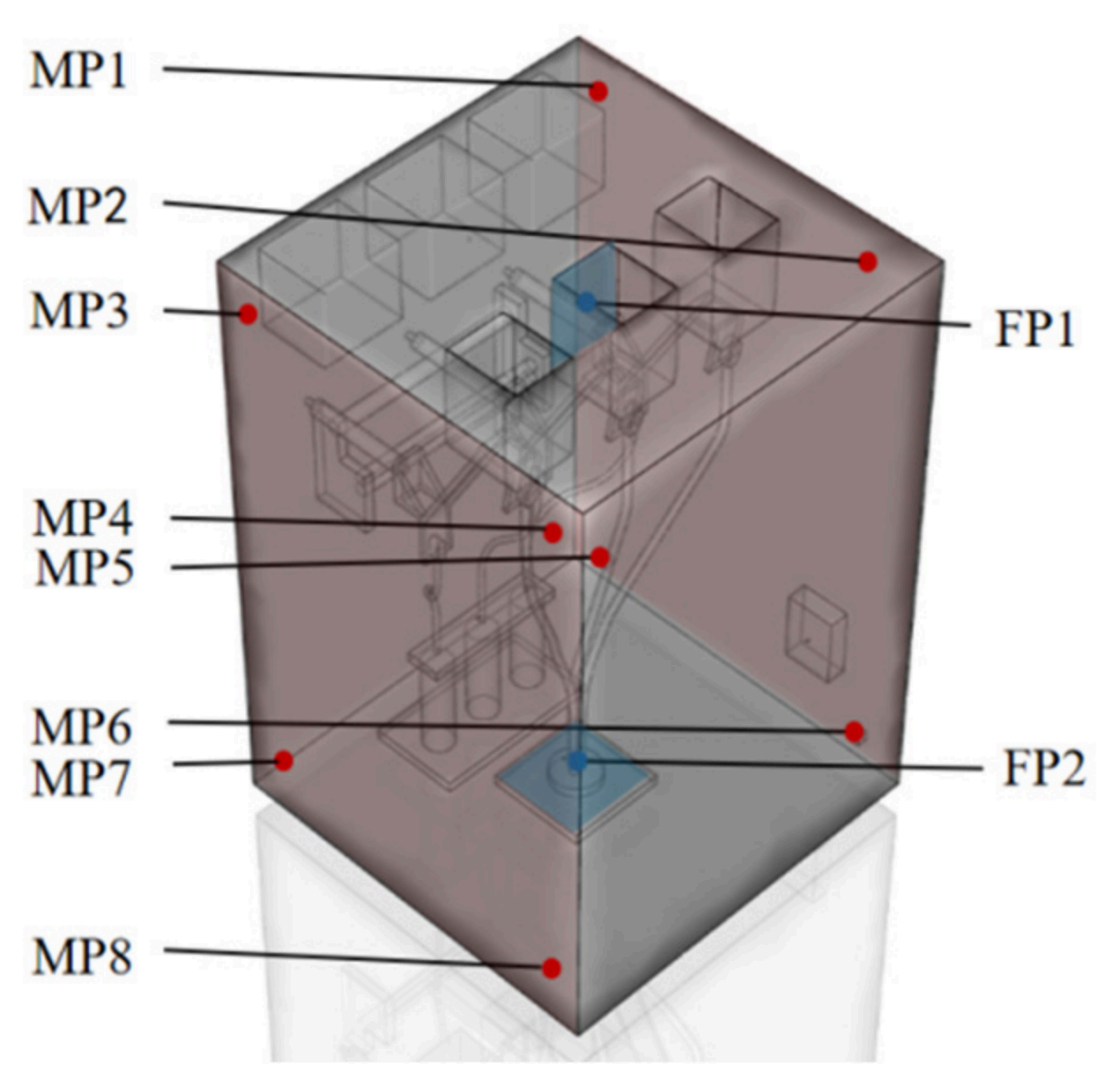
Simulation model of a high-voltage switchgear cabinet.

To verify the reliability of the numerical model, this paper has developed a full-scale simulation model of fault gas diffusion within a 10 kV switchgear cabinet. The model incorporates key structures that influence gas migration, such as the busbar compartment, contact box, cable compartment, insulating partitions, cabinet walls, and the space beneath the cabinet, to analyse the spatiotemporal evolution of fault gas diffusion from the gas generation points to the monitoring points. The resulting full-scale switchgear simulation model is shown in Figure 2, where MP1–MP8 denote monitoring points, FP1–FP2 denote fault points, and different colors represent gas concentration results under different simulation time.

Based on typical fault occurrence areas within switchgear, two types of gas generation points were established: upper and lower fault points. Upper fault points primarily correspond to faults such as partial discharge, poor contact, or insulation degradation in areas including busbar connections, circuit breaker contacts, the upper contact box and top insulation supports; lower fault points primarily correspond to overheating or discharge faults in areas such as cable terminations, the lower contact box, the bottom cable tray and earthing connection areas. In each simulation, only one fault point is activated, whilst the intensity and duration of gas release are kept consistent across different fault points to ensure comparability between different gas-generating locations.

To analyse the impact of monitoring point layout on fault gas response, a total of eight virtual monitoring points were set up on both sides of the switchgear cabinet. These monitoring points were used to simultaneously record the concentration-versus-time curves for CO, NO, CO_2_ and O_3_, and to determine the gas generation locations based on characteristics such as response time differences, peak concentration ratios and concentration integral ratios. During the tests, the release rate, release duration and monitoring point locations were kept consistent with the simulation conditions, and the reliability of the simulation model was evaluated using indicators such as initial response time, time to peak, peak concentration and concentration integral.

### 3.2. Differences in Gas Types and Physical Properties

This paper selects four gases—CO, NO, CO_2_ and O_3_—as the subjects for the analysis of fault gases within high-voltage switchgear. CO and CO_2_ originate primarily from the thermal cracking, carbonisation and oxidation of organic insulating materials; NO and O_3_ are mainly associated with air-gap discharge, corona discharge and localised ionisation processes. Differences in the molecular masses and diffusion rates of these four gases result in variations in their response speeds, migration directions and local accumulation patterns within the switchgear.

CO has a molecular mass of approximately 28.01, which is slightly lower than the average molecular mass of air; it is less affected by gravity and diffuses relatively quickly, making it suitable for early fault warning. NO has a molecular mass of approximately 30.01, which is close to that of air; its transport is primarily influenced by diffusion distance, structural obstructions, and local flow fields. CO_2_ has a molecular mass of approximately 44.01, which is significantly higher than that of air, making it more prone to localised accumulation in the lower regions. O_3_ has a molecular mass of approximately 48.00, the highest among the four gases selected in this study; its tendency to sink and remain in lower positions is more pronounced, making it more sensitive to faults in the lower regions and stagnant zones in the corners.

It should be noted that the molecular mass of a gas primarily influences its migration path, response speed, and local accumulation characteristics during the early and middle stages of diffusion. Under conditions where the enclosure is sealed, the total release volume is consistent, and diffusion is complete, the final steady-state concentrations of the same gas released from different fault points should converge. Therefore, fault localisation should focus on differences in the transient response of concentration curves, rather than relying solely on the final steady-state concentrations.

The diffusion coefficients of the four components in air (under conditions of 300 K and 0.4 Mpa) were calculated using the Fuller–Schettler–Giddings (FSG) equation [23], and the results are shown in Table 1 below:

### 3.3. Numerical Simulation Methods and Boundary Conditions

**Figure 3:**
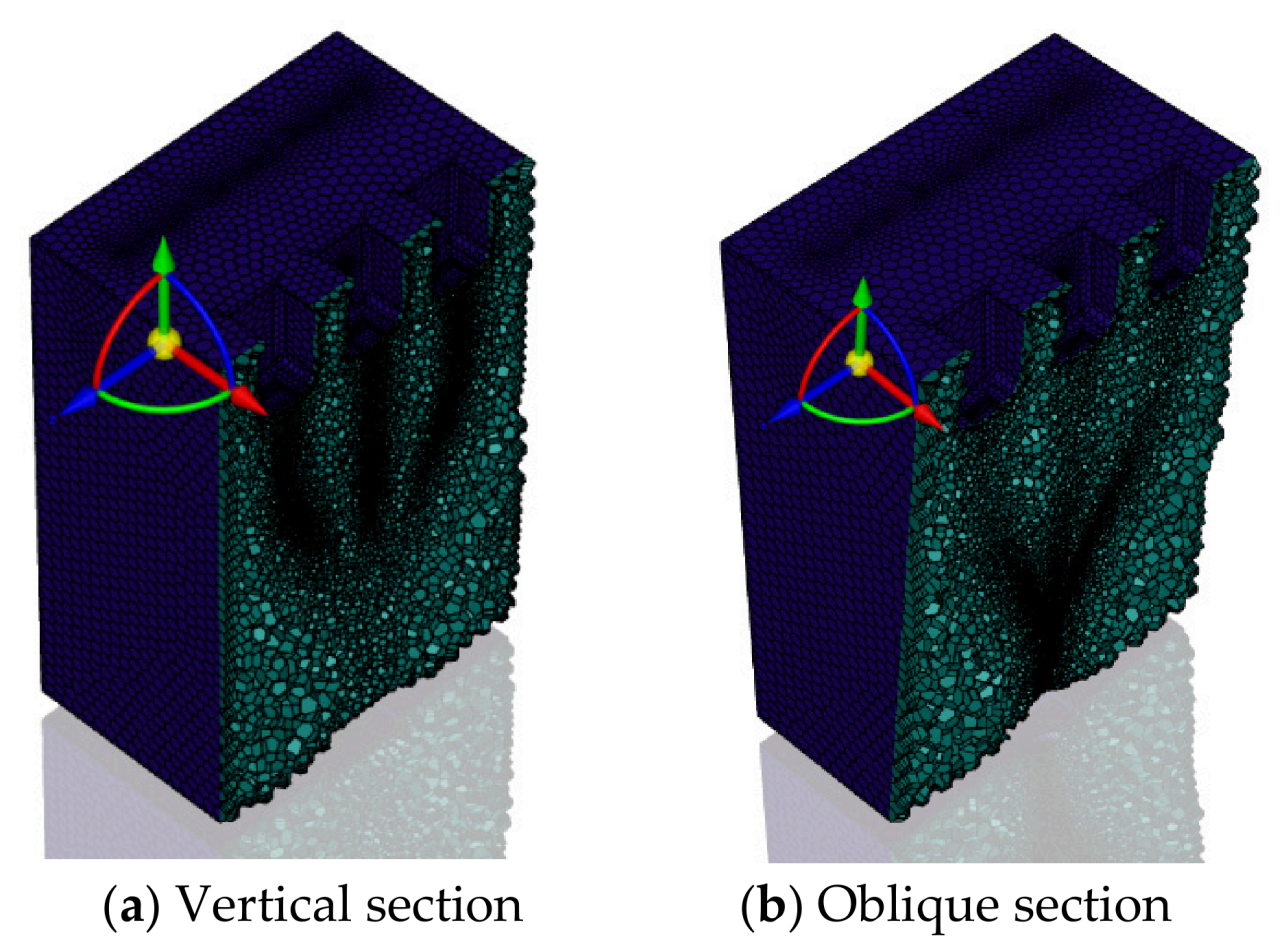
Mesh partitioning of the switchgear model surfaces.

The switchgear was meshed using the Ansys Fluent Meshing tool (ANSYS Inc., Canonsburg, PA, USA), employing a Hexcore-Polyhedral hybrid meshing strategy. A structured hexahedral mesh filled the interior of the geometry to ensure accuracy and computational efficiency; a layer of pyramids and prisms was generated between the core and the boundary to ensure a smooth transition; and polyhedral or prismatic meshes were used near the walls to accommodate complex curvatures. To ensure computational accuracy and improve the precision of gas transport calculations, local mesh refinement was applied to key areas such as the fault gas release ports, monitoring points, busbar gaps, contact boxes, cable terminations, and bottom corners. Shared topology was employed at the interface between the fluid and the solid structure within the switchgear. The mesh is shown in Figure 3.

**Figure 4:**
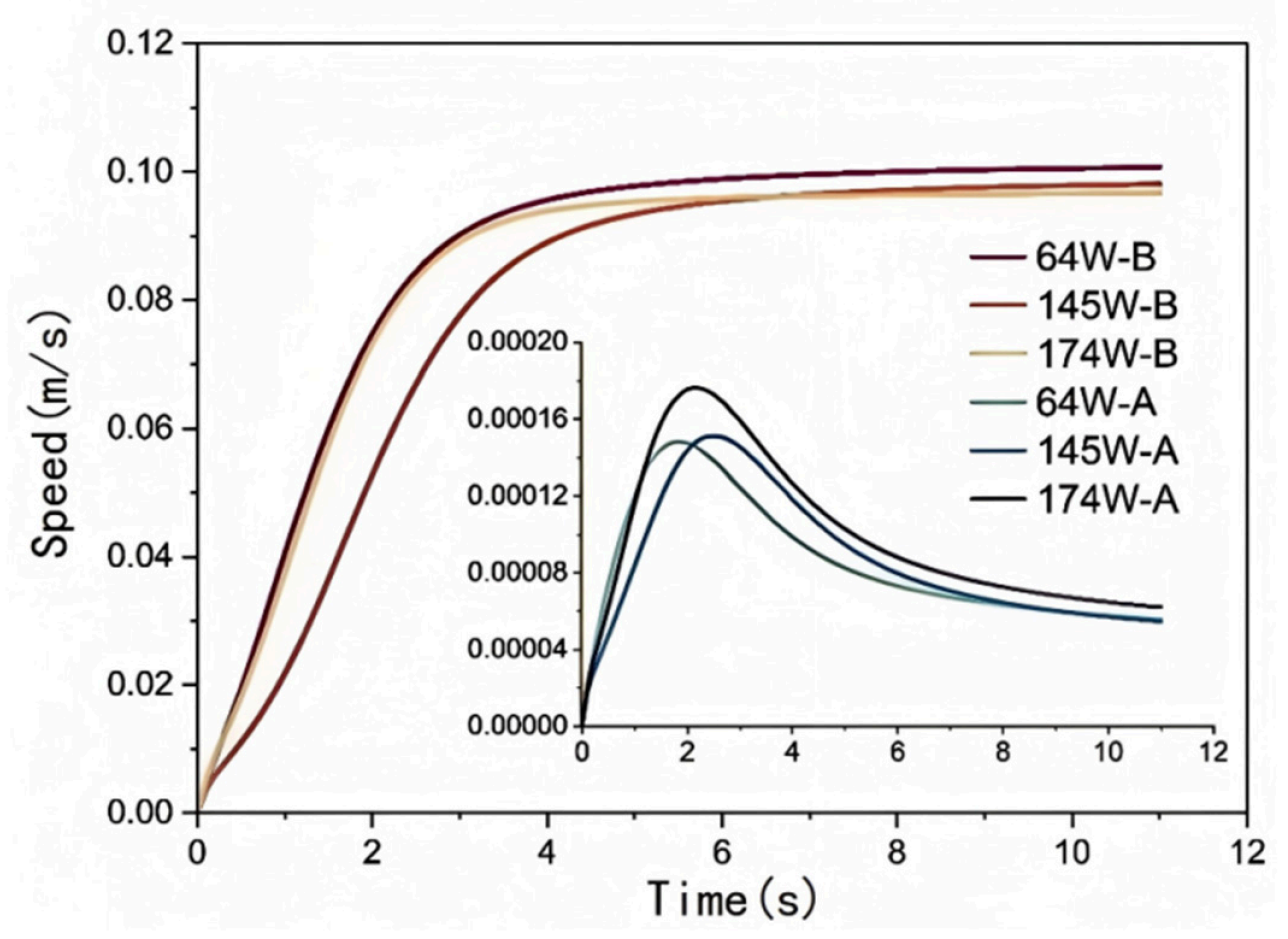
Verification of grid independence for the switchgear model.

Meshes of varying levels of precision were generated for the model, and grid independence was evaluated using three schemes containing approximately 0.64 million (64W), 1.45 million (145W) and 1.74 million (174W) cells. Figure 4 presents the velocity-related comparison. In addition, because gas concentration is the principal output used for sensing and diagnosis, the concentration–time histories at two representative monitoring points for the three meshes. The three concentration curves preserved the same response sequence and overall temporal shape; the differences in the principal concentration-response characteristics, including first response, time to peak, peak level and the late-stage concentration, remained below 10%. The mesh-dependence assessment was therefore based on both the flow field and gas-concentration response rather than on velocity alone. Considering the small change in concentration-response characteristics together with the substantially lower computational cost, the 64W mesh was selected for the subsequent transient transport calculations.

The initial pressure inside the chamber is one atmosphere and the initial gas temperature is 298 K (25 °C). For the comparative gas-transport calculations reported in this study, the isothermal transient model described in Section 3.4 is used so that the influence of source location, gas properties and internal geometry can be isolated. The cabinet is treated as a sealed domain without forced ventilation. Importantly, the geometric obstruction and channelling effects of the internal busbars, contact boxes, partitions and other structures are retained in the computational domain; only additional heat generation from these components during normal operation is neglected in the isothermal baseline. Diffusion is considered to have reached a near-uniform state when the difference between the maximum and minimum gas concentrations in the cabinet is less than 1 ppm.

This paper employs a transient gas transport calculation method to simulate the entire process of fault gas diffusion from the release point to two monitoring points within a high-voltage switchgear. During the simulation, the air inside the switchgear is treated as the background medium, and a gas source term is set at the fault point location. Only one fault point is activated per calculation, and only one type of fault gas is released, to avoid interference between different gases and to facilitate the separate analysis of the diffusion behaviour of CO, CO_2_, NO and O_3_. The same gas release intensity and release duration were applied to the upper and lower fault points, thereby ensuring comparability when evaluating the effects of different fault locations on gas transport.

The gas transport process is primarily described by the component transport equations, the basic form of which can be expressed as:

(8) $$\frac{\partial \left(\rho Y_{i}\right)}{\partial t}+\nabla \cdot \left(\rho uY_{i}\right)=\nabla \cdot \left(\rho D_{i}\nabla Y_{i}\right)+S_{i}$$

In the equation, $Y_{i}$ represents the mass fraction of the i-th gas, ρ is the density of the air mixture, u is the gas velocity vector, $D_{i}$ is the diffusion coefficient of the component, and $S_{i}$ is the gas release source term at the fault point. As there is no significant forced ventilation inside the switchgear and gas flow velocities are low, this paper focuses on the effects of molecular diffusion, weak natural convection, and the force of gravity on gas transport. For heavy gases such as carbon dioxide and ozone, the influence of the direction of gravity is particularly pronounced; whereas carbon monoxide, having a molecular mass slightly lower than that of air, tends to exhibit faster diffusion and weaker settling during its transport process.

At the beginning of each simulation, the cabinet contains background air and no additional fault gas is imposed in the bulk domain. The concentration of the standard gas introduced at the prescribed fault-source inlet is set to 1000 ppm, after which the characteristic gas is released according to the specified source condition. This value is used as a controlled reference condition for comparative transport analysis rather than as a field-typical fault concentration. Previous experimental work on 10-kV air-insulated switchgear has used an electrochemical gas sensor with a measurement range of 1000 ppm for CO, with standard-gas calibration performed before the experiment [28]. Therefore, the 1000 ppm level adopted here has a direct experimental precedent as a measurable reference level for characteristic-gas monitoring in 10-kV air-insulated switchgear. The surfaces of the cabinet, insulating components and internal structures are defined as non-slip, impermeable walls. This study focuses on the influence of fault location and gas type on monitoring-point responses; therefore, the cabinet geometry, boundary conditions, source definition and monitoring-point layout are kept identical when different gases and fault positions are compared.

### 3.4. Solution Conditions

Set the system’s reference pressure (Operating Pressure) to standard atmospheric pressure, i.e., 101,325 Pa, with the reference pressure location set at the upper surface of the switchgear model. Enable the gravity option, specifying the direction of gravitational acceleration as vertically downwards and its magnitude as 9.81 m/s^2^, and set the initial temperature to 298 K. Define the entire solution domain as a fluid domain.

Before transient fault-gas transport is simulated, a steady baseline flow field is established using a pressure-based solver. The species transport and energy equations are enabled during model setup, pressure–velocity coupling is handled using the Coupled algorithm [29], and pressure spatial discretisation uses the PRESTO! Scheme [30]. In the comparative cases discussed here, external forced ventilation and load-dependent temperature rise are not imposed; an isothermal transient species-transport calculation is then used to focus on molecular diffusion, gravity, structural obstruction and weak background convection under the controlled test condition.

A mass flow inlet boundary condition was applied at the fault injection point. As the experiment aimed to maintain a constant temperature—all heat sinks were removed during the modification of the switchgear and the outer walls of the enclosure were wrapped in thermal insulation during the test—the simulation model defined the enclosure walls as non-slip and thermally insulated, with no changes in volume or mass. Heat transfer occurs solely between the fluid and the solid within the enclosure [31].

The sealed, impermeable and thermally insulated boundaries are idealisations adopted to reproduce the controlled full-scale validation condition and to separate the gas-transport mechanism from uncertain external disturbances. Field-deployed switchgear is not perfectly sealed: cable entries, door gaps, pressure-relief structures and ventilation openings can introduce leakage or air exchange, while enclosure heat transfer and load-dependent heating can generate non-uniform temperature fields and stronger natural convection. These effects can change the absolute concentration level, dilution rate, first-arrival time and local accumulation, particularly at remote monitoring points and in stagnant zones. Consequently, the present results should be interpreted as baseline comparative transport patterns for enclosed or poorly ventilated switchgear rather than universal absolute concentration predictions. For field deployment, measured temperature/flow information, leakage or ventilation boundary conditions and calibrated fault-gas source terms should be assimilated into the digital twin.

## 4. Comparison of Simulation Results with Experimental Data

### 4.1. Evolution of Gas Concentration Fields at Different Fault Locations

To analyse the influence of the gas generation location on the spatial distribution of fault gases, transient simulations were conducted for the release of CO, NO, CO_2_ and O_3_ from both upper and lower fault points, and concentration contour plots were generated at six typical time points: 0.5 h, 1 h, 1.5 h, 2 h, 3 h and 6 h. The colour scale in the contour plots indicates increasing gas concentration from low to high, with units in ppm.

Viewed from the overall evolution process, all four gases exhibit distinct phased diffusion characteristics. In the early stage of diffusion, the gases are primarily concentrated near the fault source, forming localised high-concentration zones; as time progresses, the gases gradually diffuse into the surrounding area along structural gaps and interconnected spaces within the cabinet, with the concentration distribution range continuously expanding; in the late stage of diffusion, the concentration gradient within the cabinet gradually decreases, and the gas distribution tends towards uniformity. For a given gas, fault points located at the top and bottom of the switchgear primarily alter the position of high-concentration zones, migration paths, and the time taken to reach monitoring points during the early and middle stages of diffusion; provided that the total release volume is consistent and diffusion is thorough, the final steady-state concentrations tend to be consistent.

**Figure 5:**
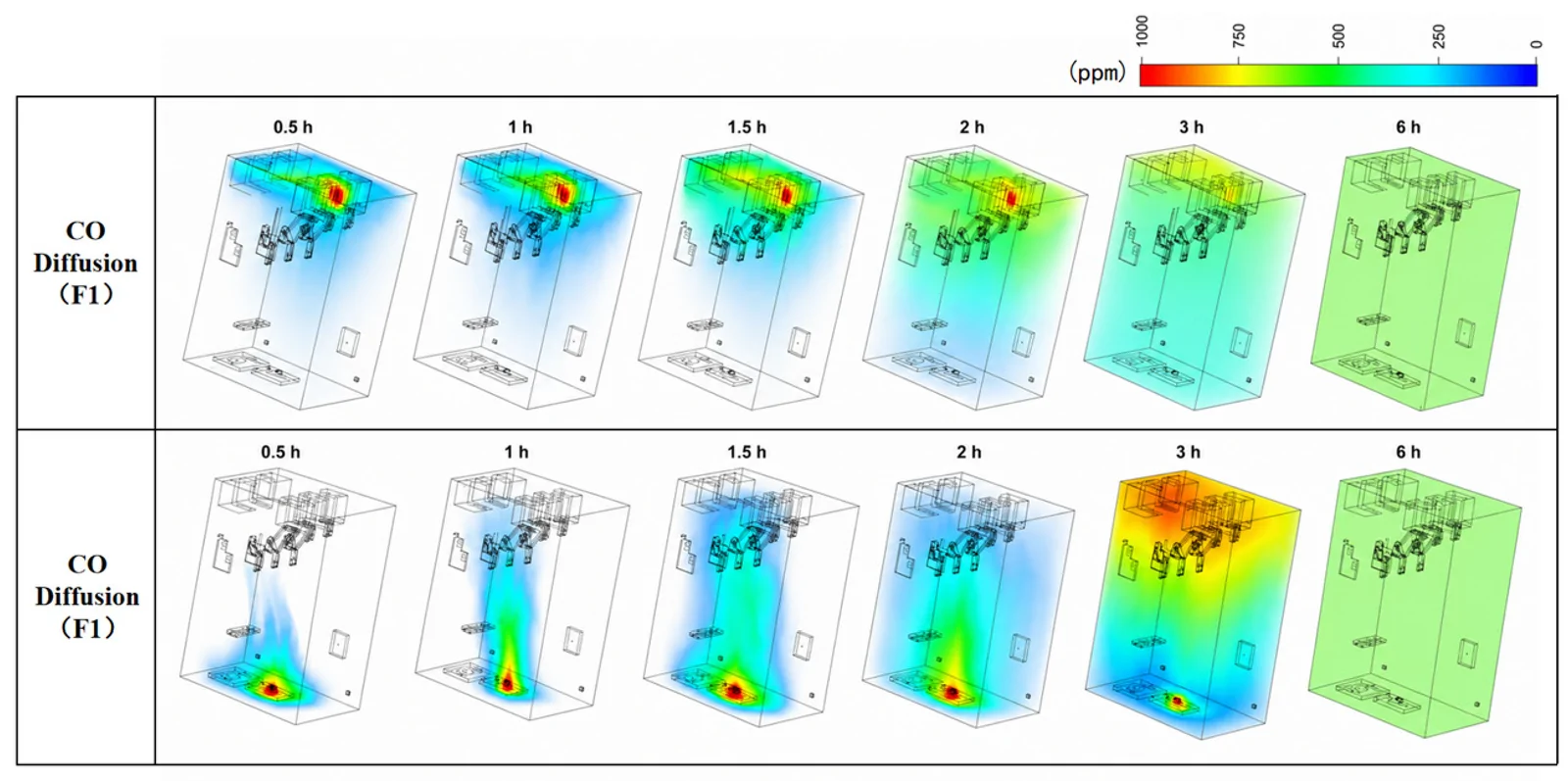
Contour map showing changes in CO concentration at two fault locations.

(1) The molecular mass of CO is slightly lower than that of air; consequently, its diffusion within the switchgear is less affected by gravitational settling, and it generally exhibits a strong capacity for spatial diffusion and a rapid tendency towards mixing. As shown in Figure 5, once CO is released from the fault source, localised high-concentration zones expand rapidly into the surrounding space, and the concentration gradient decays relatively quickly, indicating that CO is highly sensitive to gas generation in the early stages of a fault.

When a fault occurs in the upper region of the switchgear, CO first forms a high-concentration core zone near the contacts, busbar connections, or sources of partial discharge. Subsequently, the gas diffuses outward along structural gaps within the switchgear, through busbar channels, and along the edges of partition plates, gradually migrating toward the middle and lower regions of the switchgear. Because the density difference between CO and air is small, its diffusion does not show distinct vertical stratification; instead, it tends to expand in multiple directions under the influence of concentration gradients and weak convection within the switchgear. As the diffusion time increases, the upper high-concentration zone gradually expands and connects with the lower spaces, resulting in a more uniform overall concentration distribution within the cabinet.

When a fault occurs in the lower region, CO initially forms a localised enrichment zone near the fault source at the bottom; however, this high-concentration zone persists for a relatively short duration. As the diffusion process progresses, CO can escape the localised space at the bottom relatively quickly and diffuse towards the middle and upper regions. Compared with CO_2_ and O_3_, CO is less likely to remain stagnant in the lower region for extended periods; it has a greater diffusion range, and its concentration field homogenises more rapidly. This characteristic indicates that CO is more suitable for rapid response during the early stages of a fault and for early warning of abnormal gas generation.

However, CO’s strong spatial mixing capacity also diminishes the differences in concentration distribution between faults at different heights. In particular, during the later stages of diffusion, the differences in concentration cloud maps corresponding to upper and lower faults gradually diminish; relying solely on the steady-state concentration distribution of CO makes it difficult to accurately distinguish the altitude of the fault source. Therefore, when using CO for fault localisation, particular attention should be paid to the initial response time of the concentration, the rate of concentration increase, the time of peak occurrence, and the differences in response between multiple monitoring points; it is not advisable to rely solely on the concentration distribution at a single point in time as the basis for judgement.

**Figure 6:**
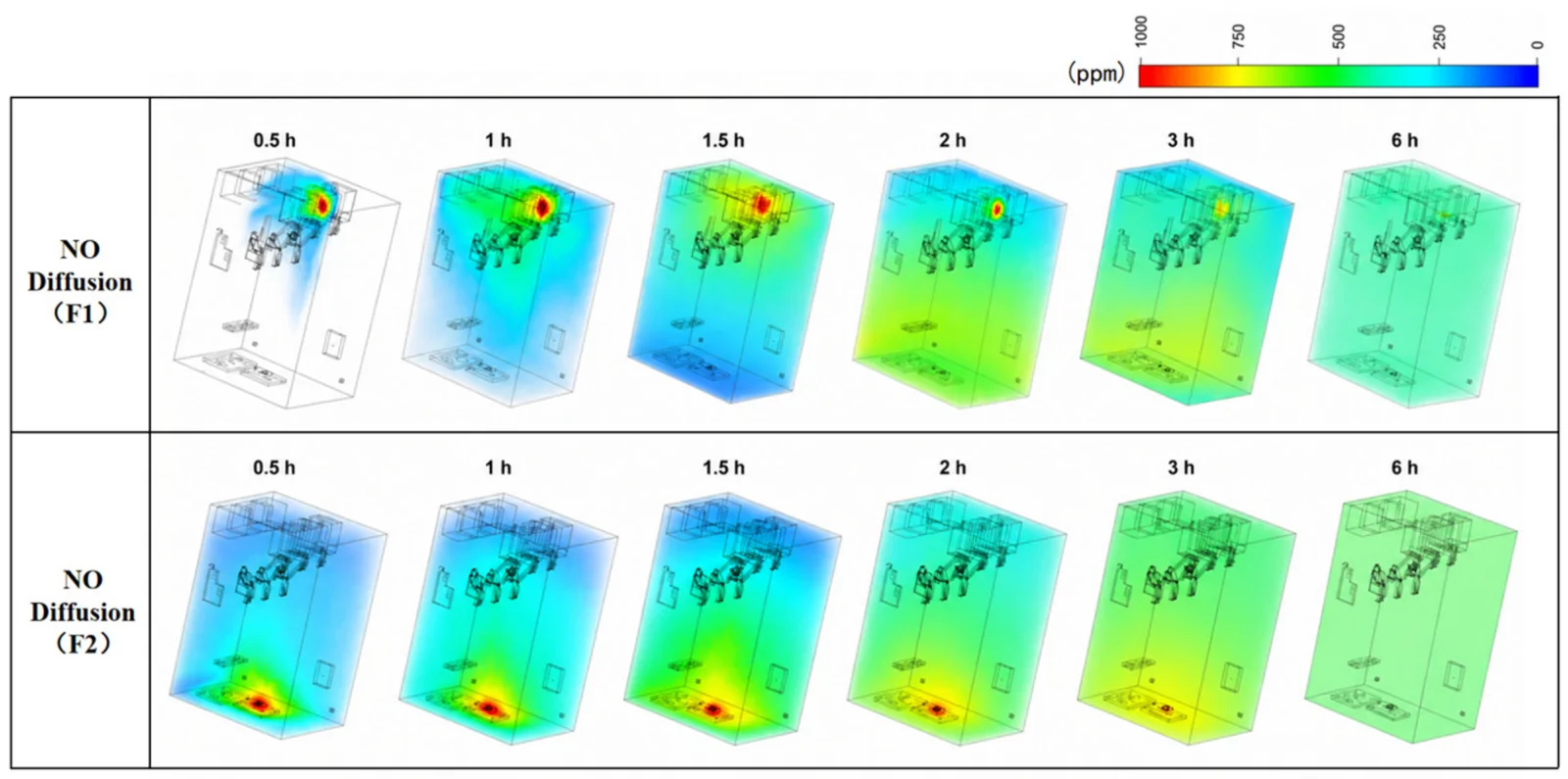
Contour plot of NO concentration variations at two fault locations.

(2) The molecular mass of NO is relatively close to that of air; consequently, it does not exhibit a pronounced tendency to rise or settle during diffusion within the switchgear. Although the molecular mass of NO is slightly greater than that of CO, this difference is insufficient to dominate its macroscopic transport direction; the evolution of its concentration field is primarily influenced by a combination of diffusion distance, structural barriers, local communication channels and natural convection within the switchgear. Consequently, the diffusion behaviour of NO more closely resembles a spatial expansion process driven by concentration gradients. The corresponding concentration-field evolution at the two fault locations is presented in Figure 6.

When the fault occurs in the upper region, NO first forms a localised high-concentration zone near the fault source. Due to the complex structures present in the upper part of the switchgear—such as contact boxes, busbars and partitions—the outward diffusion of the gas is impeded to some extent, resulting in the high-concentration zone exhibiting a distinct localised character during the initial release phase. Subsequently, NO gradually migrates towards the middle and lower regions along structural gaps and open channels; its diffusion path clearly reflects the guiding effect of the cabinet’s internal structure on gas transport. If there are localised areas of weak flow near the fault source, NO will remain stagnant for a short period before expanding to a wider spatial range in accordance with the concentration gradient.

When the fault point is located in the lower region, NO first accumulates near the fault source at the bottom, before diffusing towards the central, front and rear spaces, as well as the upper region. As NO does not exhibit the characteristic settling behaviour of heavier gases, the high-concentration zone at the bottom does not persist for long, but gradually expands to other areas within the cabinet over time. Compared with CO_2_ and O_3_, NO has a greater ability to diffuse into the upper region; however, compared with CO, its concentration field homogenises at a relatively slower rate, meaning that differences in the location of the fault source and the diffusion path can still be discerned within a certain time frame.

The main advantage of NO lies in its ability to reflect the relative distance between the fault source and the monitoring point, as well as the influence of structural passages within the switchgear on gas transport. When copper busbars, current transformers, partitions, or narrow gaps exist between the fault source and the monitoring points, the response time, concentration rise profile, and peak variations of NO are significantly affected. Consequently, NO serves as an important indicator for characterising the obstructive effects of internal cabinet structures and gas transport pathways. In fault location analysis, the time-to-first-arrival of NO, the rate of concentration rise, the peak concentration and the response delays between different monitoring points all possess significant diagnostic value.

**Figure 7:**
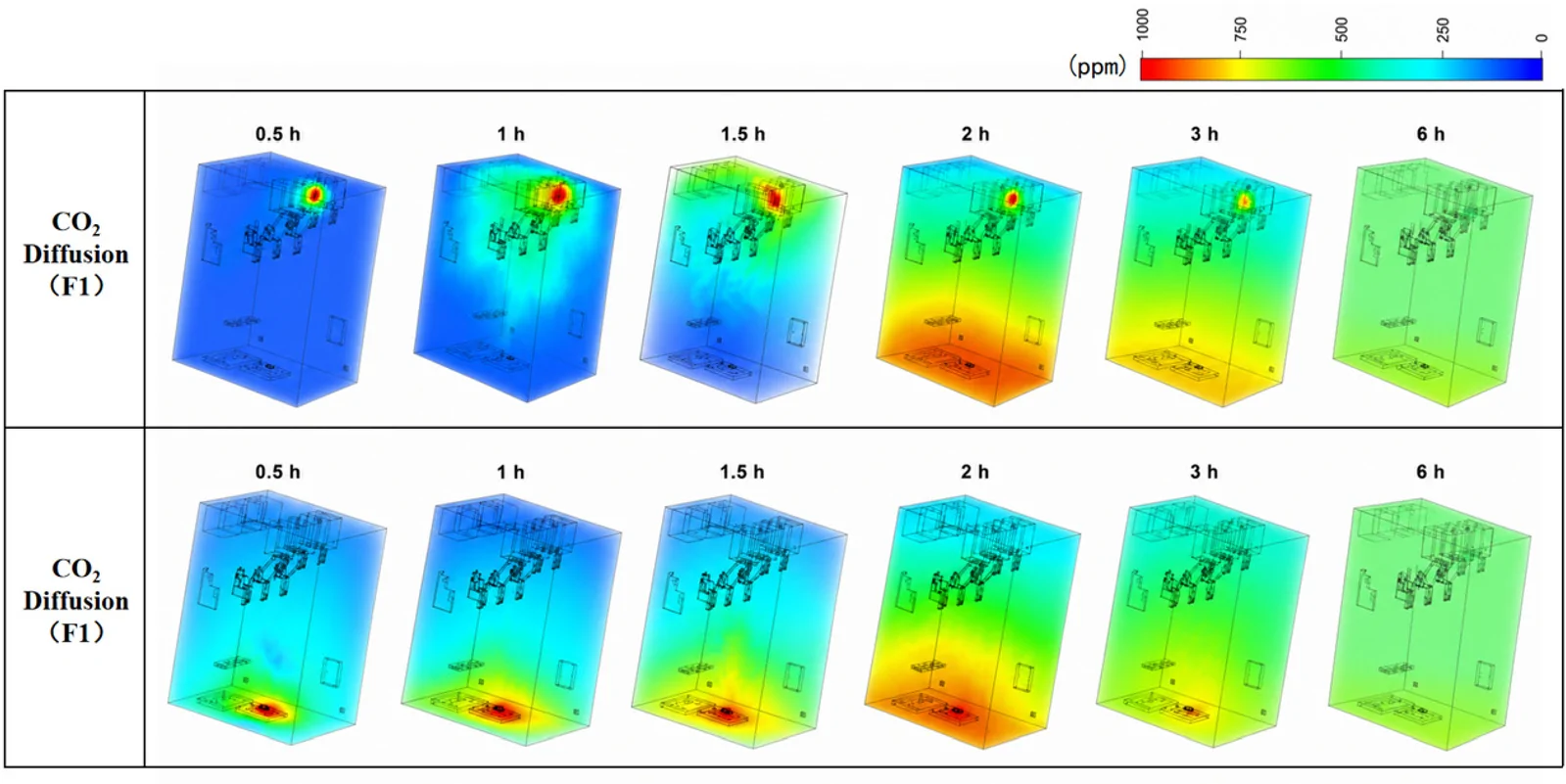
Contour plot of CO_2_ concentration variations at two fault locations.

(3) The molecular mass of CO_2_ is significantly higher than that of air; consequently, its diffusion within the switchgear exhibits the typical transport characteristics of a heavy gas. Compared with CO and NO, CO_2_ is more susceptible to gravity, tending to migrate toward lower-lying areas, spread along the bottom of the enclosure, and accumulate in regions of weak local flow during diffusion. As shown in Figure 7, the boundaries of high-concentration zones for CO_2_ are relatively distinct, its diffusion rate is slower, and spatial inhomogeneity is more pronounced.

When a fault occurs in the upper region, CO_2_ first forms a high-concentration core near the source of the fault. Subsequently, under the combined influence of concentration gradients and gravity, the CO_2_ gradually migrates towards the lower part of the enclosure. Due to the presence of copper busbars, partitions, current transformers and supporting structures within the switchgear, CO_2_ is restricted by structural passages during its downward diffusion and may accumulate near the rear wall, side walls, base plate or in localised corner areas. Compared with lighter gases, once CO_2_ has migrated to lower positions, its ability to diffuse upwards again is limited; consequently, concentration contour maps often show a pronounced tendency for enrichment at lower levels.

When a fault occurs in the lower region, the heavy-gas characteristics of CO_2_ become even more pronounced. Following gas release, a high-concentration zone first forms near the fault source at the bottom and maintains a distinct localised accumulation for an extended period. As its upward diffusion rate is relatively slow, the CO_2_ concentration field corresponding to a lower-region fault typically exhibits a distribution characterised by high concentrations at the bottom and low concentrations in the middle and upper regions. If the fault point is located in a bottom corner, a wiring area, or a local stagnation zone, CO_2_ may also exhibit more pronounced stagnation due to structural obstruction and weak convection, resulting in prolonged response times at remote monitoring points and a slower rise in concentration.

Consequently, CO_2_ exhibits high sensitivity to lower-level faults and areas of low-level accumulation. Its concentration distribution not only reflects the location of gas release but also illustrates the influence of the lower-level space within the switchgear and structural stagnation zones on the transport of heavy gases. In practical fault location, the extent of CO_2_ accumulation at the bottom, the concentration integral, the peak concentration, and the concentration ratios between different monitoring points can serve as key characteristic parameters for identifying lower-level faults and determining the height of the fault.

**Figure 8:**
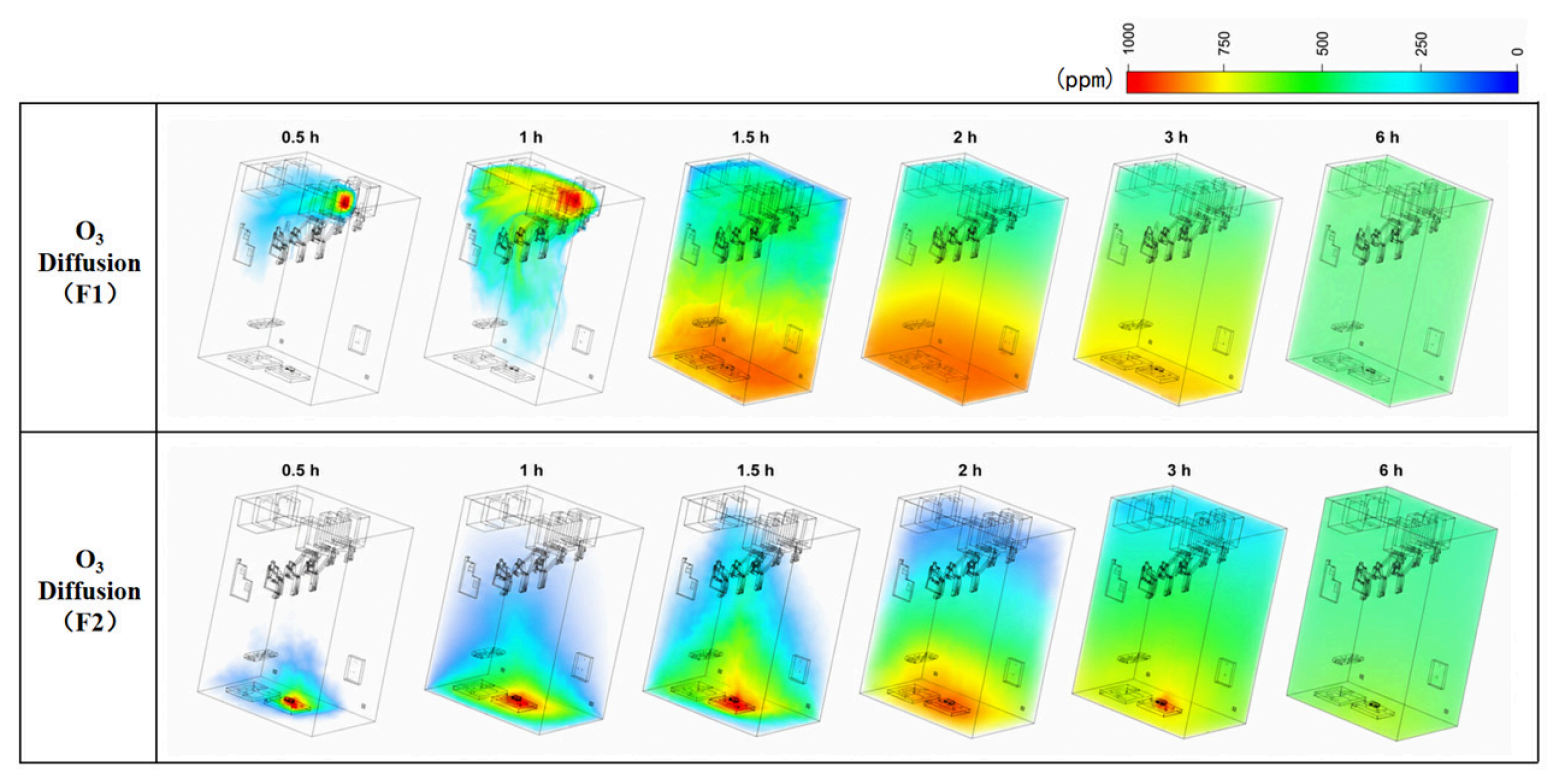
Contour plot of O_3_ concentration variations at two fault locations.

(4) O_3_ has the highest molecular mass of the four gases; consequently, its diffusion process is most significantly influenced by gravity and local flow conditions. Compared with CO and NO, O_3_ has a smaller effective diffusion range, and its concentration field homogenises more slowly; high-concentration zones are more likely to concentrate near the fault source or in low-lying areas of the cabinet with weak airflow. As shown in Figure 8, O_3_ exhibits distinct characteristics of local retention and accumulation at lower levels during diffusion, and its concentration distribution is more sensitive to changes in the location of the fault source.

When a fault occurs in the upper region, O_3_ is primarily concentrated near the fault source during the initial release phase, forming a relatively distinct high-concentration core area. Due to the relatively high molecular mass of O_3_, its diffusion rate towards distant areas is slow, and it does not readily fill the entire switchgear cabinet within a short period. As the diffusion time increases, O_3_ migrates slowly towards the middle and lower regions along structural gaps and locally connected passages. When passing through busbars, partitions, or narrow passages, the diffusion process is further restricted, meaning that the concentration cloud map retains distinct positional characteristics for a relatively long period.

When a fault occurs in the lower region, the accumulation of O_3_ at the bottom is most pronounced. Following gas release, a localised high-concentration zone rapidly forms near the fault source at the bottom and slowly expands along the base plate and gaps in the lower structural components. Owing to its limited ability to diffuse upwards, O_3_ remains in the lower region for a longer period; high-concentration zones are particularly likely to persist in the bottom wiring corners, cabinet corners and areas of low airflow. This phenomenon demonstrates that O_3_ serves as a strong spatial indicator for faults in the lower regions, corner faults, and faults in stagnant zones.

Compared with CO and NO, O_3_ has a smaller early-response range, but its high-concentration zones are more concentrated, enabling it to reflect the location of the fault source more clearly. Compared with CO_2_, O_3_ diffuses more slowly and exhibits stronger local retention characteristics; consequently, its ability to identify faults in the lower regions and corners is more pronounced. However, as O_3_ has a limited ability to be transported over long distances, if monitoring points are situated too far apart, this may result in prolonged response delays or weak concentration signals. Consequently, in practical applications, O_3_ is better suited as an auxiliary localisation gas, to be used in conjunction with gases such as CO and NO that diffuse more rapidly, to balance early fault response capability with spatial localisation accuracy.

In summary, the four gases provide complementary transient information rather than interchangeable steady-state concentration indicators. CO shows the strongest early spatial mixing and is therefore most informative through first-arrival time and early concentration-rise rate. NO is more strongly modulated by diffusion distance and structural passages, so inter-point response delay and time-to-peak are useful for identifying channelling or obstruction. CO_2_ exhibits pronounced low-level accumulation, making lower-to-upper peak and concentration-integral ratios sensitive to faults in the lower compartment. O_3_ retains localised concentration differences for longer periods and can provide additional information on low-level or stagnant-zone faults. Thus, the diagnostic value lies mainly in the early-to-middle diffusion stage, before the concentration field approaches a common late-stage distribution. Table 2 summarises these cross-gas differences and their diagnostic implications.

For practical source inference, we therefore construct the joint criterion from transient features rather than from the final concentration. For gas g at monitoring point I, the candidate feature vector contains the first-arrival time, early rise rate, time to peak, peak concentration and concentration integral. Pairwise features between monitoring points—such as the arrival-time difference, peak-concentration ratio and integral ratio—are then formed. Amplitude-related features can be normalised across monitoring points to reduce sensitivity to an unknown source strength. The measured multi-gas/multi-point feature vector can subsequently be compared with the virtual-sensor feature library generated for candidate fault regions, and candidate regions can be ranked according to their weighted discrepancy or likelihood. In this study, this framework is presented as a construction philosophy for preliminary fault-zone tracing; a fully trained real-time localisation algorithm and its independent field validation are reserved for future work.

### 4.2. Comparative Analysis of Simulation and Testing

**Figure 9:**
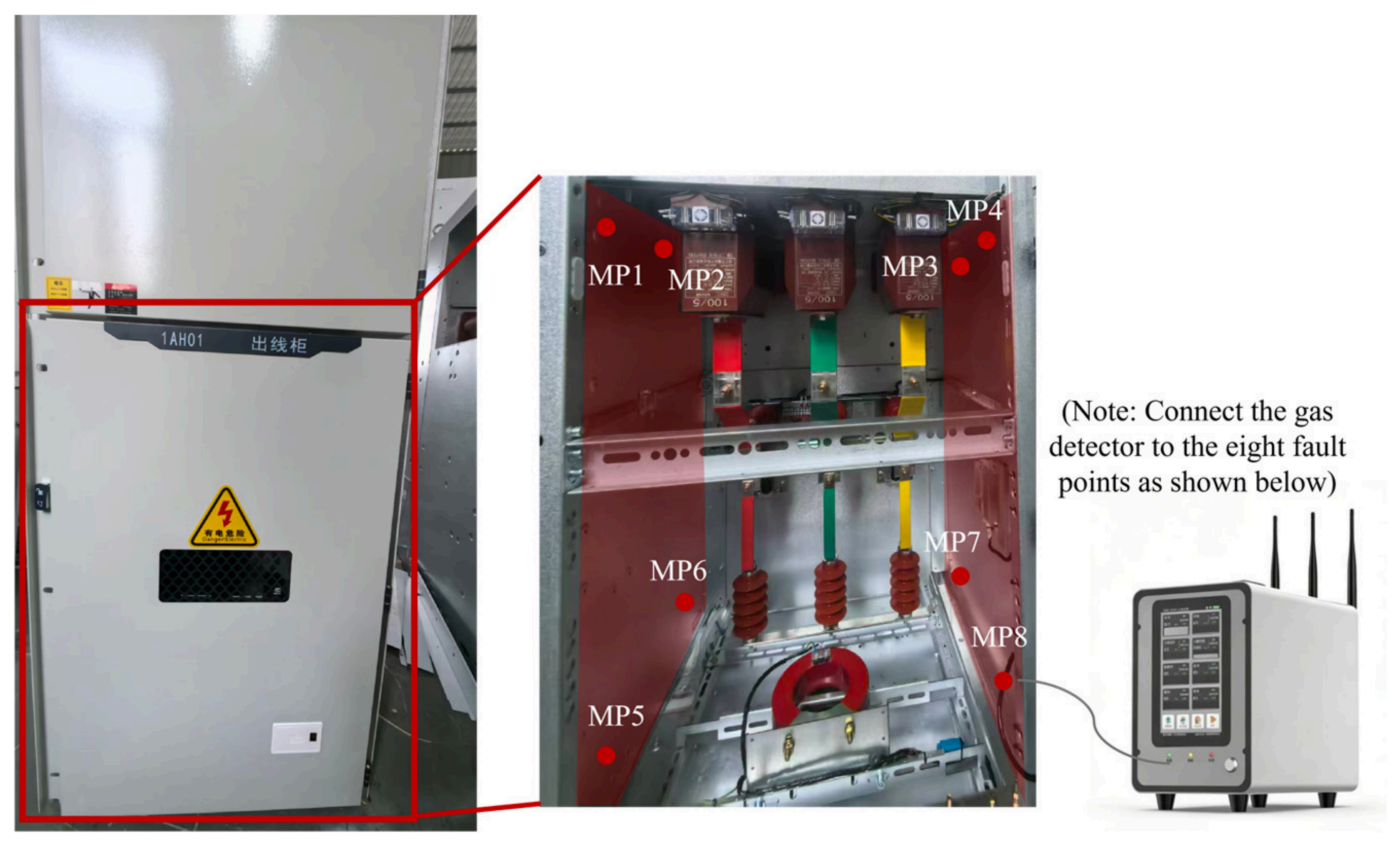
Schematic diagram of the high-voltage switchgear test bench.

The validation test was conducted on a physical 10 kV high-voltage switchgear platform as shown in Figure 9. The cabinet was sealed and filled with air at atmospheric pressure, with a nominal ambient temperature of 298 K (25 °C), to match the controlled simulation condition as closely as possible. Gas concentration was measured using a portable multi-gas analyser with built-in pump-suction sampling. Four gas-specific channels were used in the present study: electrochemical sensing channels for CO, O_3_ and NO, and an infrared-absorption channel for CO_2_. The concentration interval examined in the validation experiments was 0–1000 ppm, and the configured full-scale span of each channel covered the 1000 ppm source level. The analyser also recorded ambient temperature and relative humidity. Gas was withdrawn from the fixed sampling ports using the same sampling sequence and time interval in all comparison tests so that the measured curves could be directly aligned with the virtual-sensor histories at the corresponding locations.

The concentration interval in Table 3 denotes the range covered by the present validation data, rather than the maximum selectable full-scale range of the modular analyser. The latter depends on the installed gas-sensor module. Before each comparison, the same instrument configuration and sampling procedure were maintained for all monitoring points.

To provide a repeatable source for transport validation, characteristic gases corresponding to the numerical study (CO, CO_2_, O_3_ and NO) were introduced at representative defect locations such as conductor joints, regions near insulating supports and positions adjacent to metal protrusions. The standard-gas concentration at the source was set to 1000 ppm as a controlled reference condition rather than as a representative field-fault concentration. Previous experimental studies on air-insulated switchgear indicate that the actual concentrations of characteristic gases can vary substantially with defect type and operating conditions. For example, Gui et al. [32] reported CO and NO_2_ concentrations of up to 265 and 49 ppm, respectively, under different partial-discharge defects in an air-insulated switchgear model, although the gas analyser had measurement ranges of 0–4000 ppm for CO and 0–200 ppm for NO_2_ [32]. In another experimental study on 10-kV air-insulated switchgear, Tan et al. [28] used an electrochemical gas sensor with a measurement range of 1000 ppm for CO and calibrated the sensor using standard gas before the experiments [28]. These studies indicate that the actual fault-gas concentration is condition-dependent, while a concentration level of 1000 ppm is within the experimentally accessible measurement range for characteristic-gas monitoring.

Therefore, the 1000 ppm value adopted in the present study should not be interpreted as a universal fault concentration, alarm threshold, or typical concentration generated by all switchgear faults. Instead, it was selected as a controlled and repeatable source condition to obtain sufficiently clear transient responses and facilitate quantitative comparison between the numerical and physical models. The validation focuses on the spatial and temporal characteristics of gas transport, including the first response, concentration-rise profile, time to peak and response differences among monitoring points, rather than on reproducing a specific field-fault gas yield.

**Figure 10:**
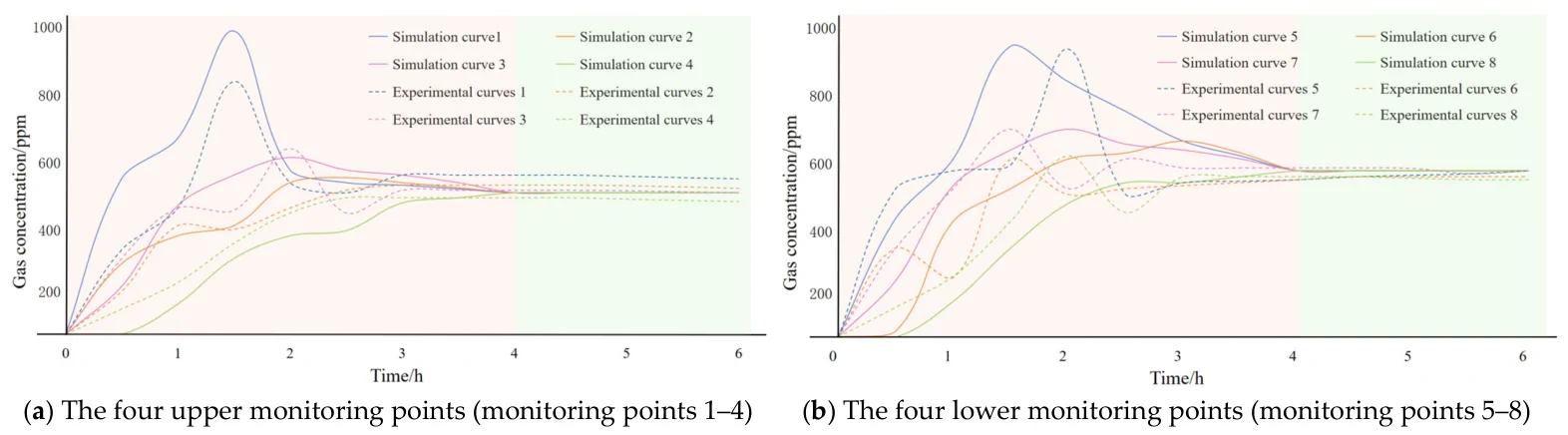
Comparison of measured data and virtual sensor results (using O_3_ gas as an example).

The measured and virtual-sensor concentration curves are compared in Figure 10; Figure 10a presents Monitoring Points 1–4, whereas Figure 10b presents Monitoring Points 5–8. The comparison in Figure 10 was performed under the controlled 1000 ppm source condition described above; therefore, the figure is intended to assess the consistency of transient transport responses between physical and virtual sensing rather than to establish 1000 ppm as a field-fault concentration.

In terms of the spatiotemporal evolution of gases, taking O_3_ as an example, its transport process exhibits distinct two-stage characteristics. During the accumulation stage (0–240 min), O_3_ molecules have a relatively high molecular mass (48.00 g/mol) and are subject to significant gravitational settling, resulting in a relatively slow diffusion rate. When a fault occurs in the upper region, O_3_ first forms a localised high-concentration core near the contact box and busbar connections. The upper sampling port closest to the injection point responds most sensitively to the rise in concentration; in particular, Monitoring Point No. 1, situated near the fault location, is the first to detect the concentration surge due to the unobstructed gas flow path between it and the release source. However, constrained by O_3_’s inherent properties as a heavy gas, its migration rate towards the lower regions is slow, resulting in a marked time lag in response between monitoring points at different heights. Concentration changes at lower monitoring points are relatively gradual, exhibiting a top-down decreasing trend in the vertical direction; furthermore, the obstruction caused by internal structural partitions and copper busbars further exacerbates this vertical disparity. Compared with other gases, CO—whose molecular mass is slightly lower than that of air—diffuses rapidly and mixes uniformly, resulting in a relatively rapid decay of the concentration gradient after release in the upper region; NO, with a density close to that of air, exhibits diffusion behaviour that is more markedly influenced by structural channels; whilst CO_2_ is also a heavy gas, O_3_ has a greater molecular mass, making it more likely to form a concentrated local enrichment zone near the release source during the early stages of diffusion, thereby providing a more pronounced spatial indication of faults in the upper region. After 240 min, the system entered a steady-state phase. As the gas release process diminished or ceased, the concentration gradient within the cabinet gradually levelled off. Under the combined effect of molecular diffusion and weak natural convection, O_3_ slowly dispersed throughout the entire cabinet; concentration differences between monitoring points gradually narrowed, and the concentration distribution within the main passageway tended towards uniformity. However, as O_3_ has the highest molecular mass, its ability to diffuse upwards is limited; even during the equilibrium phase, a certain concentration gradient may persist in the stagnant zones at the bottom and in the corners of the cabinet, reflecting the residual physical properties of heavy gases in the final mixed state. A comparison of the model simulations with the experimental data revealed that the two showed consistency in the concentration rise rate during the accumulation phase, the timing of peak occurrence, and the response timing at monitoring points; they also showed good agreement regarding the concentration decay trend during the equilibrium phase, with a maximum error in the equilibrium concentration of 9.82 per cent. This validates that the digital twin model possesses reliable simulation capabilities for such real-world physical processes and can provide effective simulation support for fault tracing and localisation based on multi-gas joint monitoring.

The comparison clarifies the respective roles of simulation and physical sensing in practical diagnosis. Physical gas sensors provide reliable time-series measurements only at a limited number of accessible ports, whereas the validated digital twin supplies virtual concentration histories and spatial fields at uninstrumented positions. For enclosed or poorly ventilated switchgear, late-stage concentrations tend to lose source-location information as the gas becomes more uniformly distributed. The diagnostic workflow should therefore emphasise the early-to-middle transient stage: measured first-arrival time, rise rate, time to peak, peak level and concentration integral are combined with inter-point time differences and concentration ratios, then compared with the corresponding virtual-sensor signatures for candidate fault regions. In this framework, CO contributes rapid early-warning information, NO helps reveal structural transport pathways, and CO_2_/O_3_ strengthen discrimination of lower- or stagnant-zone sources. Multi-gas and multi-point fusion consequently compensates for the limited sensitivity of a single gas or a single sensor position and provides a physically interpretable basis for preliminary source tracing and sensor-layout optimisation.

## 5. Conclusions

This study establishes and experimentally verifies a virtual–physical digital-twin framework for transient fault-gas transport in 10 kV air-insulated switchgear. The main conclusions and current scope are summarised as follows:

(1) Fault-source location primarily affects the early-to-middle concentration field and monitoring-point response. Upper faults initially produce high concentrations near the top and then migrate through structural gaps, whereas lower faults respond earlier at lower monitoring points and exhibit stronger local accumulation. After sufficient mixing, the concentration difference caused by source position decreases markedly; therefore, steady-state concentration alone should not be used as the primary localisation criterion.

(2) The four gases provide complementary transient signatures. Under the present controlled conditions, CO exhibits rapid early spreading and is suited to first-response warning; NO is sensitive to diffusion distance and structural channels; CO_2_ and O_3_ show stronger low-level accumulation and are more informative for lower-compartment or stagnant-zone faults. A multi-gas/multi-point criterion based on arrival-time differences, rise characteristics, peak ratios and concentration-integral ratios is therefore more informative for source tracing than a single steady-state concentration.

(3) The full-scale comparison shows good agreement between physical measurements and virtual-sensor results in concentration trend, response sequence and peak evolution, with a maximum equilibrium-concentration error of 9.82%. This agreement supports the use of the model to interpret transport paths, estimate responses at uninstrumented locations and assist sensor-layout optimisation and preliminary fault-zone tracing under conditions similar to those tested.

(4) The current study deliberately uses a sealed, impermeable and thermally insulated baseline, a controlled 1000 ppm source level supported by experimental precedent for CO measurement in 10-kV air-insulated switchgear [28,32], a limited set of fault positions and one switchgear configuration. Real equipment may exhibit leakage/ventilation, load-dependent thermal convection, variable gas-generation kinetics, sensor cross-sensitivity and chemical reactions that alter absolute concentrations and response times. Future work will incorporate measured thermal/flow boundaries and source kinetics, evaluate lower and variable concentration levels representative of specific field faults, expand the fault and cabinet database, quantify sensor uncertainty and cross-sensitivity, and develop online data-assimilation and multi-gas/multi-point probabilistic localisation algorithms for independent field validation.

## Figures and Tables

**Table 1:** Diffusion coefficients of the four components in air (at 300 K and 0.4 Mpa).

Characteristic Component Gas	Molar Mass (g/mol)	Molecular Diffusion Volume (cm^3^/mol)	Diffusion Coefficient D (m^2^/s)	Diffusion Equilibrium Time (s)
O_3_	48	26.9	1.84 × 10^−6^	1800
NO	30	39.84	1.43 × 10^−6^	2310
CO	28	46.67	1.24 × 10^−6^	2670
CO_2_	44	54.80	1.11 × 10^−6^	2960

**Table 2:** Comparative transient characteristics and diagnostic roles of the four fault gases.

Gas	Dominant Migration Response	Most Informative Transient Features	Potential Diagnostic Role
CO	Rapid early spreading; weak vertical stratification	First-arrival time; early rise rate; inter-point delay	Rapid early warning and coarse source-distance discrimination
NO	Sensitive to diffusion distance and structural passages	Inter-point delay; time-to-peak; rise profile	Identification of transport channels and structural obstruction
CO_2_	Marked low-level accumulation and slower upward transport	Lower/upper peak ratio; concentration integral; inter-point ratio	Recognition of lower-compartment and low-level accumulation faults
O_3_	Strong local retention and persistent spatial gradients	Early local peak; peak ratio; integral ratio; response delay	Auxiliary identification of low-level and stagnant-zone faults

**Table 3:** Gas-measurement configuration used for full-scale validation.

Target Gas	Sensor/Channel Type	Concentration Interval Examined	Sampling Mode
CO	Electrochemical	0–1000 ppm	Pump suction via fixed sampling port
CO_2_	Infrared absorption	0–1000 ppm	Pump suction via fixed sampling port
O_3_	Electrochemical	0–1000 ppm	Pump suction via fixed sampling port
NO	Electrochemical	0–1000 ppm	Pump suction via fixed sampling port

## Data Availability

All data that support the findings of this study are openly available.
